# Akutversorgung von Weichteilverletzungen im Kopf-Hals-Bereich

**DOI:** 10.1007/s00106-022-01231-4

**Published:** 2022-10-10

**Authors:** Amir Bolooki, Christian Offergeld, Benedikt Hofauer

**Affiliations:** 1 Klinik und Poliklinik für Hals‑, Nasen- und Ohrenheilkunde, Klinikum rechts der Isar, Technische Universität München, Ismaninger Str. 22, 81675 München, Deutschland; 2 Klinik für Hals‑, Nasen- und Ohrenheilkunde, Universitätsklinikum Freiburg, Freiburg, Deutschland

**Keywords:** Kopf-Hals-Trauma, Traumatologie, Larynxtrauma, Ohramputation, Penetrierendes Halstrauma, Head and neck trauma, Traumatology, Laryngeal trauma, Ear amputation, Penetrating neck injury

## Abstract

**Hintergrund:**

Weichteilverletzungen sind häufig Folge von Traumata im Kopf-Hals-Bereich. Einheitliche Versorgungsleitlinien zu erstellen, erweist sich als schwierig, da die Verletzungsmuster der Patienten häufig hochindividuell sind. Ziel dieser Datenerhebung war es, eine Übersicht über die Verteilung der Weichteilverletzungen zu verschaffen und die Akutversorgung der einzelnen Krankheitsbilder darzustellen.

**Material und Methoden:**

Es erfolgte eine retrospektive Auswertung anhand aller traumarelevanten ICD-10-Codes für Traumata des Kopfes (S00.- bis S09.-) und des Halses (S10.- bis S19.-), die in einem Zeitraum von zehn Jahren (2012 bis einschließlich 2021) an unserem Klinikum, einem zertifizierten überregionalen Traumazentrum, behandelt wurden.

**Ergebnisse:**

Insgesamt wurden im Beobachtungszeitraum 8375 Patienten mit Traumata des Kopfes und Halses versorgt, also durchschnittlich 836 Patienten jährlich. Innerhalb dieses Kollektivs wurden 2981 Trauma mit Weichteilverletzungen dokumentiert. Oberflächliche Verletzungen des Kopfes (S00.-) und offene Wunden des Kopfes (S01.-) waren mit 1649 bzw. 920 Fällen die häufigsten Weichteilverletzungen des Kopf-Hals-Bereichs.

**Schlussfolgerung:**

Die Fallzahlen der Weichteilverletzungen haben in der Regel einen inversen Zusammenhang zum benötigten zugrunde liegenden Trauma. Diagnosen der Kategorie S00 und S01 kommen deshalb häufiger vor als beispielsweise traumatische Amputationen in Halshöhe (S18). Penetrierende Traumata des Halses sollten gemäß aktueller Literatur nach einem sog. No-Zone-Prinzip versorgt werden. Aufgrund der niedrigen Kriminalitätsraten und strengen Waffenschutzgesetze sind Verletzungen solcher Art in Europa eher selten.

## Abstract (en)

**Background:**

Soft tissue injuries are a common consequence of head and neck trauma. With injuries being highly individual and varying depending on the underlying trauma, it is difficult to establish standardized guidelines for head and neck trauma in general. The main goal of this study was to showcase the distribution of soft tissue injury types and the principles pertaining to acute care of the individual clinical presentations.

**Materials and methods:**

A retrospective evaluation was carried out using all trauma-relevant ICD-10 codes for trauma to the head (S00.- to S09.-) and neck (S10.- to S19.-) among patients who were treated at the authors’ clinic—a certified national trauma center—during a period of 10 years (2012 to and including 2021).

**Results:**

A total of 8375 patients with head and neck trauma were treated during the observation period, i.e., an average of 836 patients per year. Within this collective, 2981 trauma cases involving soft tissue injuries were documented. Superficial injuries to the head (S00.-) and open wounds to the head (S01.-) were the most common head and neck soft tissue injuries, with 1649 and 920 cases, respectively.

**Conclusion:**

The case numbers of soft tissue injuries generally show an inverse correlation to the required underlying trauma: diagnoses of the categories S00 and S01 occur very often; injuries which only occur after severe trauma, such as traumatic amputation at neck level (S18), are rare. According to current literature, penetrating neck traumas should be treated using a no-zone approach. In Europe, penetrating neck injuries are rather rare because of low crime rates and strict weapon laws.

## Hintergrund

Traumata des Kopf-Hals-Bereichs können eine große Bandbreite an Folgen nach sich ziehen. Eine schnelle und gute Erstversorgung ist ausschlaggebend für den weiteren Krankheitsverlauf. Ziel ist es, mögliche Komplikationen zu minimieren oder sogar komplett abzuwenden. Unfälle mit Bezug zum Kopf-Hals-Bereich können zu Verletzungen von Knochen und/oder Weichteilen führen. Diese können sich, je nach Art und Schwere des zugrunde liegenden Traumas, als ein komplexes klinisches Bild multipler Verletzungen unterschiedlicher Schwere präsentieren. Häufig kann das eigentliche Ausmaß der Verletzungen nicht auf den ersten Blick, sondern erst im Rahmen der klinischen Versorgung, unterstützt mit entsprechenden diagnostischen Methoden, erfasst werden. Hierzu zählt eine ausführliche Anamnese, insbesondere den Unfallhergang betreffend, als auch eine systematische traumaorientierte Untersuchung aller Strukturen des Kopf-Hals-Bereichs. Anschließend wird dies durch – dem Unfallhergang angemessene – bildgebende Verfahren komplettiert. Eine wichtige Rolle spielt die interdisziplinäre Zusammenarbeit verschiedener Fachabteilungen zur adäquaten Einschätzung der Verletzungen, um ein dem Patienten individuell zugeschnittenes Therapiekonzept zu finden. Zudem sollte immer eine zeitnahe Versorgung angestrebt werden, da sich diese positiv auf das Outcome der Patienten auswirkt. Ziel der Datenerhebung war es einen Überblick über die Fallzahlen traumabedingter Verletzungen im Kopf-Hals-Bereich zu erstellen und hierbei besonders das Verteilungsmuster der verschiedenen Weichteilverletzungen darzustellen. Ergänzt werden die erhobenen Daten durch eine Erläuterung zur Akutversorgung der einzelnen Weichteilverletzungen [1–5].

## Material und Methoden

Im Rahmen der durchgeführten retrospektiven Studie wurden die Fallzahlen der an der Klinik und Poliklinik für Hals‑, Nasen- und Ohrenheilkunde des Klinikums rechts der Isar der Technischen Universität München versorgten Patienten mit Traumata im Kopf-Hals-Bereich untersucht. Die Versorgung von Unfallpatienten findet innerhalb eines zertifizierten überregionalen Traumazentrums statt, wobei unser Traumazentrum den zentralen Anlaufpunkt des Trauma-Netzwerks München/Oberbayern Nord darstellt. Diese Region beinhaltet neben ländlichen Gebiete auch mehrere größere Verkehrswege/Autobahnen sowie städtische Areale. Erfasst wurden die Fälle der letzten zehn Jahre (2012 bis einschließlich 2021) mithilfe der entsprechenden ICD-10-Codes (Tab. 1 und 2).

**Table 1:** 

S00.- Oberflächliche Verletzung des Kopfes
S01.- Offene Wunde des Kopfes
S02.- Fraktur des Schädels und der Gesichtsschädelknochen
S03.- Luxation, Verstauchung und Zerrung von Gelenken und Bändern des Kopfes
S04.- Verletzung von Hirnnerven
S05.- Verletzung des Auges und der Orbita
S06.- Intrakranielle Verletzung
S07.- Zerquetschen des Kopfes
S08.- Traumatische Amputation von Teilen des Kopfes
S09.- Sonstige und nicht näher bezeichnete Verletzungen des Kopfes

**Table 2:** 

S10.- Oberflächliche Verletzung des Halses
S11.- Offene Wunde des Halses
S12.- Fraktur im Bereich des Halses
S13.- Luxation, Verstauchung und Zerrung von Gelenken und Bändern in Halshöhe
S14.- Verletzung der Nerven und des Rückenmarkes in Halshöhe
S15.- Verletzung von Blutgefäßen in Halshöhe
S16.- Verletzung von Muskeln und Sehnen in Halshöhe
S17.- Zerquetschung des Halses
S18.- Traumatische Amputation in Halshöhe
S19.- Sonstige und nicht näher bezeichnete Verletzungen des Halses

In der weiteren detaillierten Auswertung wurde das Hauptaugenmerk, basierend auf der Häufigkeit und der Relevanz der Fälle, auf ausgewählte Verletzungen gelegt (Tab. 3).

**Table 3:** 

S00.- Oberflächliche Verletzung des Kopfes
S01.- Offene Wunde des Kopfes
S08.- Traumatische Amputation von Teilen des Kopfes
S10.- Oberflächliche Verletzung des Halses
S11.- Offene Wunde des Halses
S15.- bis einschließlich S19.-

## Ergebnisse

**Figure 1:**
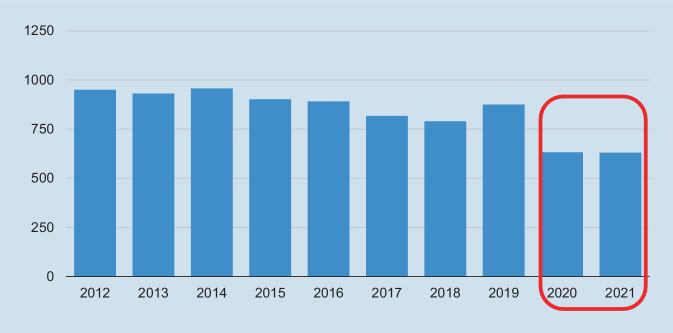

Es stellten sich im Beobachtungszeitraum jährlich durchschnittlich 836 Patienten mit traumabedingten Verletzungen im Kopf-Hals-Bereich vor, wobei 2014 mit insgesamt 957 Fällen ein Höchstwert erreicht wurde. Mit jeweils nur knapp 630 Fällen stellten sich in den Jahren 2020 und 2021 deutlich weniger Patienten mit traumabedingten Verletzungen vor. Die häufigste Diagnose waren Nasenbeinfrakturen (S02.‑) mit durchschnittlichen 406 Fällen pro Jahr und einem Anteil von 41 % an allen erfassten Traumata (Abb. 1 und 2).

**Figure 2:**
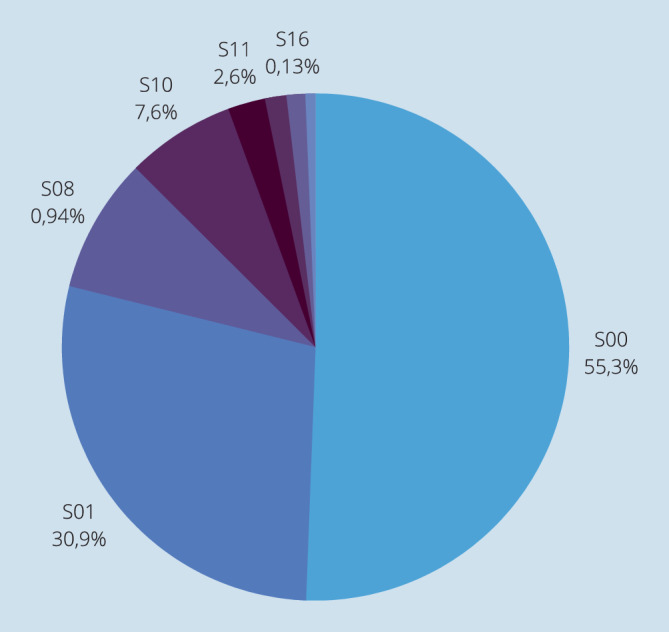

Insgesamt machten Weichteilverletzungen mit 2981 von insgesamt 8375 Fällen des Beobachtungszeitraums von zehn Jahren 36 % der Kopf-Hals-Traumata aus. Die häufigsten Weichteilverletzung hierbei waren oberflächliche Verletzungen des Kopfes (S00.-) mit 1649 Fällen (Schürfwunden und andere Wunden, die nicht mittels Wundnaht versorgt werden mussten). Verletzungsmuster der Kategorie S17.- (Zerquetschen des Halses) und S18.- (Traumatische Amputation in Halshöhe) traten nicht auf (Tab. 4).

**Table 4:** 

Kopfverletzungen ICD-10-Code	Gesamtanzahl	Halsverletzungen ICD-10-Code	Gesamtanzahl
S00.-	1649	S10.-	229
S01.-	924	S11.-	80
S02.-	4049	S12.-	96
S03.-	25	S13.-	15
S04.-	8	S14.-	1
S05.-	14	S15.-	46
S06.-	265	S16.-	4
S07.-	0	S17.-	0
S08.-	28	S18.-	0
S09.-	921	S19.-	21

## Diskussion

Im Folgenden werden die zu den unterschiedlichen Lokalisationen erhobenen Daten zusammen mit allgemeinen und lokalisationsspezifischen Behandlungsempfehlungen diskutiert.

### Grundlegende Maßnahmen in der Wundversorgung

Unabhängig von Traumalokalisation und -schwere gibt es grundlegende Maßnahmen, die sofort ergriffen werden sollten. Es ist eine zeitnahe Wundversorgung anzustreben, um das Komplikationsrisiko zu minimieren und ästhetischen Ansprüchen im Kopf-Hals-Bereich gerecht zu werden. Neben einer Reinigung der Wunde kann abhängig von Größe und Kontamination auch eine Spülung angezeigt sein, wobei diese entweder mit isotonischer Kochsalzlösung, bei Bedarf auch mit einer Mischung aus isotonischer Kochsalzlösung und Povidon-Iod erfolgen kann [6, 7]. Die Wundspülung sollte jedoch keinesfalls mit Antiseptika erfolgen, die nur für eine oberflächliche Anwendung bestimmt sind (wie z. B. Octenidin), da sonst das Risiko toxischer Gewebeeffekte besteht. Anschließend sollte, falls nötig, ein Débridement erfolgen, um optimale Wundverhältnisse für die darauffolgende Wundheilung zu schaffen, wobei gerade im Gesichtsbereich nur so wenig wie möglich debridiert werden sollte. Eine Antibiotikaprophylaxe gegen Wundinfekte sollte immer individuell abgewogen werden, ist jedoch bei immunkompetenten PatientInnen und sauberen Wundverhältnissen nicht erforderlich [8, 9].

Darüber hinaus muss im Rahmen der Anamnese der aktuelle Tetanusimpfstatus eruiert werden. Orientiert an der Art der Wunde und dem Impfstatus der Patienten kann eine akute Immunisierung notwendig sein [10]. Die Art des Wundverschlusses richtet sich nach Lokalisation, Schwere und Unfallhergang [11]. Viele der hier erfassten Weichteilverletzungen sind bereits unter diesen Maßnahmen vollständig versorgt. Hierzu zählen zahlreiche der unter S00, S01 und S11 gelistete Diagnosen.

Die oberflächlichen Verletzungen des Kopfes (S00.-) umfassen durchschnittlich 165 Fälle pro Jahr. Passend zu den bereits erwähnten Fallzahlen der Nasenbeinfrakturen, ist die häufigste Diagnose für S00.- eine Prellung der Nase. Zum Ausschluss einer möglichen Fraktur ist es wichtig, eine gezielte körperliche Untersuchung durchzuführen. Bereits Mithilfe von Inspektion, Palpation und vorderer Rhinoskopie lassen sich viele Prellungen von Frakturen abgrenzen. Je nach Unfallhergang und Begleitverletzungen wird die Indikation für bildgebende Verfahren gestellt. Bei Verdacht auf Komplikationen und schwere Begleitverletzungen sollte eine Computertomographie (CT) durchgeführt werden. Bei reinen Prellungen reicht eine symptomatische Therapie, bestehend aus Analgesie und Kühlung der Nase [12].

Offene Wunden des Kopfes (S01.-) umfassen 92 Fälle pro Jahr. Diese zeigen keine eindeutige Häufung bestimmte Bereiche des Kopfes betreffend. Offene Wunden des Halses sind mit 8 Fällen pro Jahr relativ selten.

### Traumatischer Abriss des Ohrs

**Figure 3:**
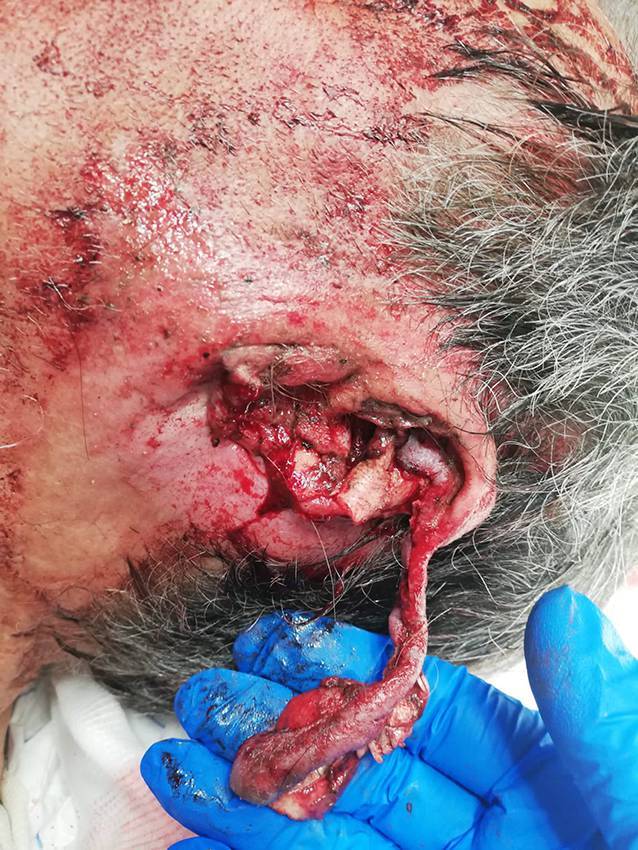

Amputationen der Ohrmuschel sind häufig Folge von Verkehrsunfällen und Tätigkeitsdelikten. Mit drei Fällen pro Jahr treten sie in unserer Klinik relativ selten auf. Die Art der chirurgischen Versorgung richtet sich nach der Schwere der Verletzung. Zunächst werden die genannten Basismaßnahmen ergriffen. Besonders ein ausgiebiges Débridement verschmutzter und verletzter Knorpel- und Hautareale ist wichtig. Man differenziert zwischen subtotalen und totalen Amputationen (Abb. 3).

Subtotale Ohramputationen sind dadurch charakterisiert, dass das abgerissene Stück durch einen Hautpedikel weiterhin Kontakt zum restlichen Ohr hat. Dieser Stiel gewährleistet eine gewisse Durchblutung, weshalb derartige Amputationen häufig mithilfe von einfachen Nähten wieder adaptiert werden können.

**Figure 4:**
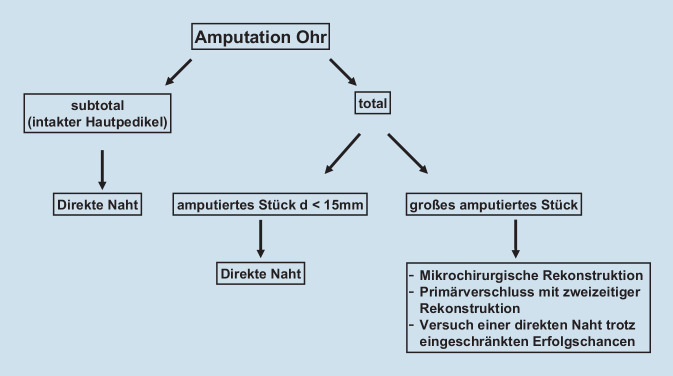

Totale Amputationen hingegen stellen eine größere Herausforderung dar. Eine direkte Naht des amputierten Stücks wird empfohlen, wenn dieses einen Durchmesser von < 15 mm hat. Bei größeren Defekten ist die Wahrscheinlichkeit einer Nekrose im Rahmen einer direkten Naht erhöht. Eine Alternative bietet eine mikrochirurgische Rekonstruktion. Vorteil dieser Methode ist die Gewährleistung einer adäquaten Blutversorgung durch eine Anastomosierung der Gefäße. Zudem liefert sie ausgezeichnete ästhetische Ergebnisse. Jedoch ist eine derartige Rekonstruktion nicht immer möglich, da je nach Unfallhergang nicht immer adäquate Gefäße zur Verfügung stehen. Eine Alternative zu einer einzeitigen definitiven Versorgung bieten mehrzeitige operative Konzepte. Im Rahmen der Erstvorstellung kann durch ein Débridement so viel vitales Gewebe wie möglich gerettet und die Wunde primär verschlossen werden. Im Verlauf kann dann eine endgültige Rekonstruktion stattfinden [13–18]. Ein Beispiel für ein zweischrittiges Vorgehen ist die Replantationsmethode nach Baudet, bei der die Rückfläche der Ohrmuschel denudiert und gefenstert wird und so auf eine Wundfläche auf dem Mastoid, die dort durch Entfernung der Haut geschaffen wird, eingenäht werden kann. Nach drei Monaten kann die Ohrmuschel angehoben und die beiden Hautdefekte (Rückseite Ohrmuschel und Mastoid) mit Vollhaut gedeckt werden [19, 20]. Wenn die natürliche Ohrmuschel nicht mehr erhalten werden kann, dann ist eine Rekonstruktion, etwa mit Rippenknorpel, oder eine Epithesenversorgung möglich. In Abb. 4 werden die Versorgungsmöglichkeiten zusammengefasst.

### Akutes Larynxtrauma

**Figure 5:**
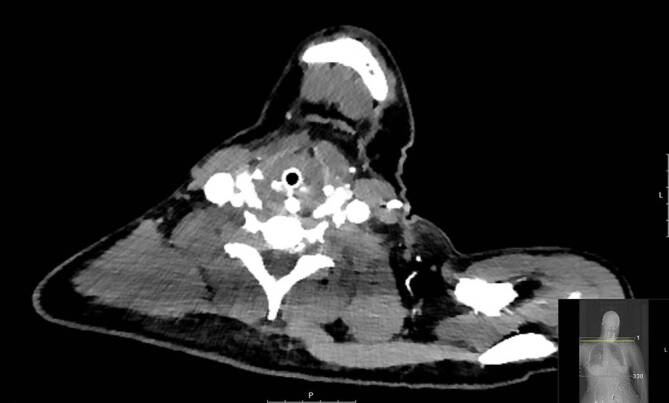

Zu den oberflächlichen Verletzungen des Halses (S10.-) zählen Prellungen des Larynx, der Trachea und der Pars cervicalis des Ösophagus. Häufige Folgen sind Einblutungen in die Halsweichteile und endolaryngeale Schwellungen. Im Schnitt verzeichnen wir in unserer Klinik ungefähr 23 entsprechende Fälle pro Jahr. Zervikale Traumata sind sehr heikle Krankheitsbilder, da von außen häufig keine schwerwiegenden Unfallfolgen imponieren. Die Patienten stellen sich mit teils harmlos scheinenden Symptomen wie Dysphonie und Dysphagie vor oder zeigen gar keine Symptome. Prellmarken sind nur gelegentlich sichtbar. Endolaryngeal kann es jedoch durch eine Ödembildung zu einer kompletten Verlegung der Atemwege kommen (Abb. 5).

Oberste Priorität hat die Sicherung der Atemwege. Instabile Atemwege müssen sofort durch Intubation oder durch eine Tracheotomie gesichert werden. Erst anschließend sollte das Ausmaß der Verletzungen durch weitere diagnostische Schritte abgeklärt werden.

Zur adäquaten Befunderhebung sollte eine flexible Endoskopie der oberen Atemwege durchgeführt werden, um Einblutungen und Ödeme schnell zu erkennen. Bei allen Patienten mit einer Prellung des Halses sollte eine CT mit Angiographie zum Ausschluss von Gefäß- und Larynxverletzungen durchgeführt werden. Selbst bei klinisch und CT-morphologisch unauffälligen Befunden ist mit den Patienten eine Aufnahme zur stationären Überwachung zu diskutieren, da es auch 24–48 h nach dem initialen Trauma zur Ödementwicklung kommen kann. Therapeutisch kann mithilfe einer Steroidgabe der Ödementwicklung entgegenwirkt werden. Die weiteren Schritte richten sich nach den Begleitverletzungen [21–23]. Bei Larynxfrakturen mit klinisch relevanter Dislokation der Fragmente sollte eine zeitnahe Versorgung erfolgen, abhängig vom Ausmaß der Dislokation endoskopisch oder über einen offenen Zugang. Die Chondrosynthese kann über Nähte oder über Mikroplatten erfolgen und in aller Regel ist eine begleitende Schutztracheotomie erforderlich.

### Penetrierende Kopf- und Halstrauma

**Figure 6:**
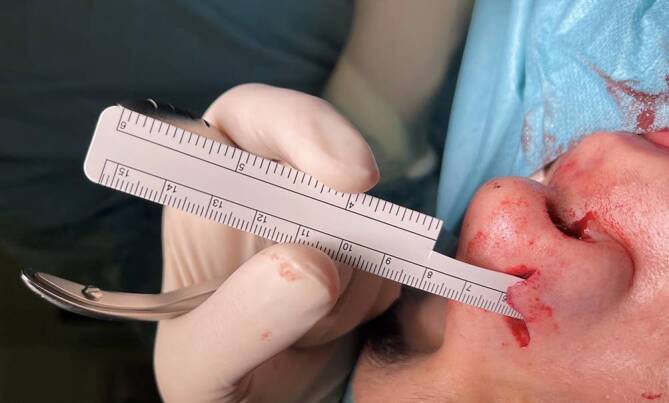

Penetrierende Kopf- und Halsverletzungen sind fulminante Krankheitsbilder mit diversen zugrunde liegenden Unfallmechanismen. Häufige Auslöser sind Gewaltverbrechen (Schussverletzungen, Messerstiche) oder Motorradunfälle (Abb. 6).

Die Mortalität ist höher als bei stumpfen Halstraumata. Die Herangehensweise orientierte sich an den 3 definierten Halszonen (nach Monson) [24]:

- Zone 1: Klavikula bis Ringknorpel
- Zone 2: Ringknorpel bis Kieferwinkel
- Zone 3: Kieferwinkel bis Schädelbasis

Penetrierende Halstraumata betreffen am häufigsten Zone 2. Eine Beteiligung essenzieller Strukturen wird in der Regel durch ein intaktes Platysma ausgeschlossen. Um unnötige Explorationen zu vermeiden, fand über die Jahre ein Übergang zu einer No-Zone-Herangehensweise statt, bei dem das Ausmaß der Verletzungen bei stabilen Patienten erst im Rahmen einer CT-Untersuchung mit Angiographie evaluiert und nur bei vorliegenden „hard signs“ ein sofortiges operatives Vorgehen gewählt wurde. Zur Kategorie der „hard signs“ gehören unter anderem [25]:

- schwerwiegende aktive Blutung,
- Stroke-Symptomatik,
- großes progredientes Hämatom,
- Beteiligung der Atemwege,
- subkutanes Emphysem.

Diese neue Herangehensweise ist vor allem dahingehend sinnvoll, dass penetrierende Halstraumata sich selten auf nur eine Halszone beschränken. Vaskuläre Verletzungen sind die häufigsten Komplikationen penetrierender Halstraumata. Je nach betroffener Halszone kommt es zur Beteiligung weiterer Organsysteme (Trachea, Ösophagus, Halsnerven).

Initial hat die Sicherung des Atemwegs die höchste Priorität. Bei hämodynamischer Instabilität oder dem Vorliegen von „hard signs“ sollte eine zeitnahe chirurgische Exploration erfolgen. Abgesehen davon bietet es sich jedoch an mithilfe einer CT mit Angiographie das Ausmaß der Verletzungen zu evaluieren und anschließend zielgerichtet weitere diagnostische oder therapeutische Schritte einzuleiten. Aufgrund der Nähe verschiedenster anatomischer Strukturen im Halsbereich ist eine Zusammenarbeit diverser Fachdisziplinen notwendig, um die Traumata adäquat zu versorgen. Die weiteren therapeutischen Schritte reichen je nach Ausmaß der Verletzungen von konservativen Therapiekonzepten bis hin zu aufwendigen Rekonstruktionen der Atemwege, Speisewege und vaskulären Strukturen [25–31].

Betrachtet man die Kopf-Hals-Traumata der letzten zehn Jahre, zeigt sich eine Reduktion der Fallzahlen für die Jahre 2020 und 2021. Diese Reduktion lässt sich am ehesten auf die Verhaltensregeln und weiteren Regelungen (Kontaktbeschränkungen, weniger Alkoholkonsum, deutliche Beschränkungen des Nachtlebens) seit Beginn der COVID-19-Pandemie zurückführen. Ein derartiger Zusammenhang in Bezug auf Traumata generell wurde bereits mehrmals in der Literatur beschrieben [32].

Betrachtet man die Fallzahlen der Weichteilverletzungen, zeigt sich, dass leichtgradige Weichteilverletzungen wesentlich häufiger als schwergradige auftreten. So wurden in unserer Klinik im Beobachtungszeitraum keine Fälle von traumatischer Amputation in Halshöhe (S18.-) oder Zerquetschen des Halses (S17.-) verschlüsselt. Es ist zudem anzumerken, dass derartige Verletzungen mit einer hohen Mortalität noch am Unfallort einhergehen, wodurch häufig keine weitere innerklinische Versorgung durchgeführt wird.

Verletzungen des Halses zeigen im Verhältnis zu Verletzungen des Gesichts geringe Fallzahlen und treten seltener isoliert, sondern häufiger in Kombination mit weiteren Verletzungen auf.

Trotz harmloser äußerer Traumafolgen im Bereich des Halses sollte immer eine Evaluation der endolaryngealen Strukturen erfolgen, um Schwellungen früh zu erkennen und entsprechend reagieren zu können. Eine geringe Symptomatik bei Erstvorstellung kann dazu führen, dass keine weitere Diagnostik oder stationäre Überwachung in die Wege geleitet wird. Hier sollte jedoch unbedingt die Gefahr einer Ödembildung auch noch nach 24–48 h berücksichtigt werden. Die Prävention einer akuten Verlegung der oberen Atemwege hat höchste Priorität [22].

Penetrierende Halstraumata sind, wie in vielen anderen europäischen Ländern, eher selten. Ursächlich sind die strengen Waffenschutzgesetze. Zudem korrelieren penetrierende Halstraumata durch z. B. Messerstich- oder Schussverletzungen mit der allgemeinen Kriminalitätsrate der entsprechenden Länder [28].

Als Limitation dieser Untersuchung ist anzumerken, dass die Auswertung basierend auf ICD-10-Codes immer von der Qualität der Codierung abhängig ist. Gerade bei multiplen simultanen Verletzungen, wie sie bei Traumata häufig auftreten, wird häufig nur die führende Diagnose verschlüsselt, sodass möglicherweise kleinere Begleitverletzungen nicht abgebildet werden. Zudem kann man durch die Verwendung unspezifischer Codes die genaue Traumafolge nicht spezifizieren [33]. Dennoch dient die Auswertung einer orientierenden Darstellung der Häufigkeitsverteilung der Weichteilverletzungen. Vor allem die Diagnosen mit den größten Fallzahlen sind auch unter Berücksichtigung des Untersucher-Bias repräsentativ für die Verteilung von Verletzungsmustern in anderen Kliniken.

## Fazit für die Praxis

- In einem Drittel aller traumatologischer Fälle des Kopf-Hals-Bereichs kommt es zu Weichteilverletzungen.
- Die Mehrheit der Weichteilverletzungen stellen oberflächliche Verletzungen dar, tiefergehende und fulminantere Verletzungen sind verhältnismäßig selten.
- Neben Basismaßnahmen, die für alle Verletzungslokalisation einheitlich Gültigkeit haben, unterscheiden sich lokalisationsspezifische Maßnahmen teilweise deutlich.
- Auch bei diskreten äußerlichen Verletzungszeichen können vital bedrohliche innere Verletzungen vorliegen – eine entsprechende diagnostische Aufarbeitung ist daher zwingend geboten.
